# Psychometrics Properties of Early Trauma Inventory Self Report – Short Form (ETISR-SR) for the Brazilian Context

**DOI:** 10.1371/journal.pone.0076337

**Published:** 2013-10-03

**Authors:** Flávia L. Osório, Giovanni Abrahão Salum, Mariana Fortunata Donadon, Larissa Forni-dos-Santos, Sonia Regina Loureiro, José Alexandre S. Crippa

**Affiliations:** 1 Department of Neurosciences and Behavior – Medical School of Ribeirão Preto - University of São Paulo, Ribeirão Preto, Brazil; 2 Technology Institute (INCT, CNPq) for Translational Medicine, Ribeirão Preto, Brazil; 3 National Institute of Developmental Psychiatry for Children and Adolescents - CNPq, São Paulo, Brazil; 4 Federal University of Rio Grande do Sul, Rio Grande do, Federative Republic of Brazil, Sul, Brazil; Federal University of Rio de Janeiro, Brazil

## Abstract

This study aims to translate and validate *Early Trauma Inventory Self Report -Short Form* (*ETISR-SF*) to Brazilian Portuguese. 253 adult subjects answered the ETISR-SF, Beck Anxiety Inventory (BAI), Fagerström Test for Nicotine Dependence (FTND), Patient Health Questionnaire (PHQ-9) and Fast Alcohol Screening Test (FAST). The instrument showed good internal consistency (0.83). Correlations with the PHQ-9 and BAI were moderate (r=0.26-0.47) and showed the expected associations with psychiatric constructs. No associations were found for FTND and FAST. Confirmatory Factor Analysis revealed that a correlated four-factor model as well as a second order model subsuming four lower order components presented the best model fit. Test-retest reliability was also excellent (ICC=0.78-0.90). ETISR-SF is suitable for assessing traumatic experiences in a Brazilian community sample. Given the importance of trauma as a public health problem, tools such as ETISR-SF may help clinicians/ researchers to better evaluate and measure such events and further advance clinical care of trauma victims.

## Introduction

Early traumatic experiences are highly prevalent [1,2] and constitute risk factors for atypical development [3]. They have a significant impact on the biological, social emotional and cognitive functioning of the individual, and also favor the emergence of psychiatric disorders, whether in infancy or in adulthood [3-8]. They are also associated with significant financial costs regarding medical treatment and psychosocial and social services [9]. Proper assessment and measurement of traumatic experiences in childhood and adolescence is critical for both clinical and research purposes, especially in low and middle income countries where such measures are lacking.

The best way to access this information is not agreed upon in the literature, with the evaluation proposition verified through open and/or structured interviews, as a form of hetero-evaluation of the history of the trauma, and through self-evaluation instruments. A literature review indicated a variety of instruments developed for this purpose. Among them, the following hetero-applied instruments can be underscored: *Traumatic Antecedents Interview* [10] *Retrospective Assessment of Traumatic Experience* [11], *Child Experience of Care and Abuse* [12], *Childhood Trauma Interview* [13] *and Early Trauma Inventory* [14]. Regarding the self-applied instruments in the national and international literature podemos ilustrar alguns deles: *Assessing Environments III* [15], *Childhood Trauma Questionnaire* [16] *Trauma History Questionnaire* [17] and the short version and of the self administered *Early Trauma Inventory* [18]. In the context of Brazil, few instruments for such purpose have been validated [19].

The *Early Trauma Inventory Self Report – Short Form* (*ETISR-SF*) was originally proposed by Bremner et al. [18]. It is comprised of 27 items, divided into four dimensions (general trauma, physical abuse, emotional abuse and sexual abuse) and scored on a dichotomous scale (Yes/No). It was evaluated for validity and reliability, demonstrating adequate indicators. Further studies were conducted with this scale in different languages (Spanish, Chinese, Korean and Dutch) and with different samples (drug dependent subjects, war veterans, depressive patients and puerperae) [20-24], which again indicated the adequacy of the scale, endorsing and encouraging its use in different cultures.

The aim of the present study was to translate and validate the *Early Trauma Inventory Self Report – Short Form* (*ETISR-SF*) scale for the Brazilian context, studying the following psychometric properties: internal consistency, concurrent/divergent and discriminative validity, factor structure, and test-retest reliability.

## Materials and Methods

### 1: Study Sample

The study sample consisted of 253 subjects: university students (N= 60), healthcare, education and cleaning workers (N=60), primary care patients (N=53), and inpatients of a general hospital (N=80). They were selected by convenience and their participation was voluntary.

### 2: Ethics Statement

All ethical requirements related to research with human beings were respected, with approval given by the site Ethics Committee for the performance of the study (Hospital das Clínicas da Faculdade de Medicina de Ribeirão Preto - USP - Protocol No. 2316/2011). All subjects gave written informed consent after being fully informed of the research procedure.

### 3: Instruments

a Early Trauma Inventory Self Report – Short Form (ETISR-SF) - the object instrument of this study, which was translated and adapted into Brazilian Portuguese. It is comprised of 27 items, divided into four dimensions (general trauma, physical abuse, emotional abuse and sexual abuse) and scored on a dichotomous scale (Yes/No).
b Beck Anxiety Inventory (BAI) - developed by Beck and Steer [25] and translated, adapted, and validated for the Brazilian Population by Cunha [26]. This self-administered instrument consists of 21 items that evaluate the severity of anxiety symptoms, which are scored on a Likert scale between 0 and 4.
c Fagerström Test for Nicotine Dependence (FTND) – elaborated by Karl-Olov Fagerström in 1978 [27], this is a self-rating instrument consisting of six items that intend to measure the degree of physical nicotine dependence. The version translated into Brazilian Portuguese by Carmo & Pueyo [28] was used in the present study.
d Patient Health Questionnaire (PHQ-9) – a self-rating instrument consisting of nine items based on the criteria of major depression proposed by the Diagnostic and Statistical Manual of Mental Disorders, 4th edition (DSM-IV). The version translated and validated for Brazilian Portuguese was used in the present study [29].
e Fast Alcohol Screening Test (FAST) – a self-rating instrument for the assessment of the risky and harmful use of alcohol and alcohol dependence syndrome. The version translated and validated for Brazilian Portuguese by Meneses-Gaya et al [30] was used in the present study.

### 4: Procedures and Data Analysis

The ETISR-SF was translated and adapted to Brazilian in accordance with standardized technical recommendations [31]. The instrument was first translated from its original English version into Portuguese by an experienced translator and was then independently translated by two Brazilian psychiatrists with good knowledge of the English language. The three versions were compared and discussed by two bilingual raters with wide experience in psychiatric evaluation scales, who performed the verification of semantic equivalence and, after reaching a consensus, proposed a translated version of the scale. This version was then independently back translated by a bilingual psychiatrist, who had no access to the original English version, and presented to the author of the original scale for appreciation. The author did not suggest any modification, considering the version of the scale to be adequate, and formally authorized the official use of this Portuguese version.

In order to further establish the adequacy of the instrument, four Brazilian psychiatrists with substantial experience in the use of scales acted as raters, evaluating the instrument in terms of item pertinence and formulation, confirming its face validity. Pilot testing with a reduced number of subjects (N=20) was performed in order to determine the semantic understanding of the instructions and of the item formulation. No suggestions of modifications to be incorporated into the final version of the instrument were made. This step was thus considered to conclude the translation and adaptation stage of the ETISR-SF (Figure S1).

The data were collected individually by a professional trained in the application of scales. The subjects received a booklet containing the instruments described above and the evaluator was available to clarify any doubts. The data were organized into a database and submitted to statistical analysis using the SPSS software, version 13.0 (2001; SPSS Inc, Chicago, Ill). The demographic data of the sample under study were analyzed by applying descriptive and non parametric statistical tests.

The following procedures were applied for the study of validity of the ETISR-SF:

• Cronbach’s α for the evaluation of the internal consistency of the scales. The α values considered to be acceptable were those exceeding .60 [32,33];
• Spearman’s correlation coefficient (r) for the total score and the scales ETISR-SF, BAI, FAST, FTND and PHQ-9 to assess the concurrent/divergent validity between scales. The magnitude of the correlations detected was defined as follows: 0 to 0.25, weak; 0.26 to 0.50 moderate; 0.51 to 0.70 strong; and above 0.71 very strong [31];
• Mann Whitney test for the analysis of discriminative validity as a function of the presence or absence of indicators of psychiatric symptoms (depression, anxiety and abuse substance);

The confirmatory factor analysis (CFA) models were fitted to polychoric correlations among the ETIS-SR items using mean- and variance-adjusted weighted least squares (WLSMV) estimator implemented with Mplus 7.0 [34], given the categorical nature of the items. We tested four specific models: (a) a single trauma factor as a result of the 27 items; (b) a correlated four factor scale (general, physical, emotional and sexual) – proposed by the original version; (c) a second-order model, with one high order factor (trauma) as a result of four lower order factors (general, physical, emotional and sexual); (d) a bifactor (or hierarchical) model, in which all items load in a general trauma latent factor and residual variances explained by four specific factors. The goodness of fit was assessed through the following fit indices: chi-square, WRMR (weighted root mean square residual), CFI (comparative fit index), TLI (Tucker-Lewis Index) and RMSEA, (root mean square error of approximation). To demonstrate good fit to the data, an estimated model should have a WRMR near or bellow 0.9 [34], and RMSEA of near or bellow 0.06 and CFI and TLI near or above 0.95 [35].

• The intraclass correlation coefficient was used for the study of inter-rater reliability [31].

The level of significance was set at p ≤ 0.05 in all analyses.

## Results

### 1: Sociodemographic characterization of the sample

The sample consisted of 253 adult subjects of both genders (male = 56.1%) aged between 21 and 71 years (mean=40.88; standard deviation=14.87), with high school education prevalent among the varied education levels (46,2%).

### 2: Analysis of Items

The score frequency of each item of the scale was evaluated and is presented in Table 1, together with the mean and standard deviation values.

**Table 1:** Frequency, mean and standard deviation of the different items of the ETISR-SF.

**Subscale**	**Item**	**F (%)**	**Mean (SD)**
**General Traumas**	1.1. Natural disasters	19 (7.5)	0.08 (0.26)
	1.2. Serious accident	35 (13.8)	0.14 (0.35)
	1.3. Injury/ Illness	38 (15.0)	0.15 (0.36)
	1.4. Death/ Illness parents	60 (23.7)	0.24 (0.43)
	1.5. Divorce parents	62 (15.8)	0.29 (0.67)
	1.6. Death/ Injury siblings	40 (20.2)	0.16 (0.37)
	1.7. Death/ Injury friend	47 (18.6)	0.19 (0.39)
	1.8. Violence situations	74 (29.2)	0.29 (0.46)
	1.9. Mental disorder in the family	61 (24.1)	0.24 (0.43)
	1.10. Alcohol/ Drug Use Parents	55 (21.7)	0.22 (0.41)
	1.11. Murder	42 (16.6)	0.17 (0.37)
	Total	-----	2.09 (1.91)
**Physical Abuse**	2.1. Slap in the face	73 (28.9)	0.29 (0.46)
	2.2. Burn water/ cigarette	27 (10.7)	0.11 (0.31)
	2.3. Punch/ Kick	71 (28.1)	0.28 (0.45)
	2.4. Thrown object	68 (26.9)	0.27 (0.44)
	2.5. Pushed	124 (49.0)	0.49 (0.50)
	Total	-----	1.43 (1.47)
**Emotional Abuse**	3.1. Ridiculed	60 (23.7)	0.24 (0.43)
	3.2. Ignored	53 (20.9)	0.21 (0.41)
	3.3. Told that you were no good	32 (12.6)	0.13 (0.33)
	3.4. Lack of affection / Love	46 (18.2)	0.18 (0.39)
	3.5. Parents did not understand the needs	59 (23.3)	0.23 (0.42)
	Total	-----	1.00 (1.54)
**Sexual Abuse**	4.1. Touching body parts	38 (15.0)	0.15 (0.36)
	4.2. Rubbing genitals	39 (15.4)	0.15 (0.36)
	4.3. Touching intimate parts of another	17 (6.7)	0.07 (0.25)
	4.4. Sex against your will	15 (5.9)	0.06 (0.24)
	4.5. Oral sex	7 (5.9)	0.03 (0.16)
	4.6. Sexualized kiss	10 (4.0)	0.04 (0.20)
	Total	-----	0.49 (1.04)

It can be observed that the highest-rated items were associated with physical abuse. Among the general traumas, there are the experiences of violence against themselves or family members, and divorce of the parents. Among the emotional traumas highlighted are the difficulty of the parents to understand and meet the personal needs of the child and experiences of ridicule. Among the sexual traumas, events where someone rubbed their genitals against the child and touched body parts predominated. Evaluating the presence of trauma by category, it can be seen that approximately 75.5% of the sample experienced some type of general trauma, 60.5% physical abuse, 39.1% emotional abuse and 24.1% sexual event/abuse.

### 3: Internal Consistency

The alpha value for the total scale was calculated and found to be 0.83. Concerning the individual subscales, the alpha of 0.54 was found for general trauma, 0.69 for physical abuse, 0.83 for emotional abuse and 0.73 for sexual events. The items correlated with the total with values ranging from 0.13 (item 1.3 – “Did you ever suffer a serious personal injury or illness?’) to 0.60 (item 3.4 - “Most of the time were you treated in a cold, uncaring way or made to feel like you were not loved?”). None of the items would alter the value of alpha if removed from the scale.

### 4: Concurrent and Divergent Validity

The study of the concurrent/divergence validity of the scale was conducted through correlation with other measures that evaluate constructs correlated to the experiences of early trauma, as indicated by the literature [1,4,7,8], namely: depression, substance abuse and anxiety. The data are presented in Table 2.

**Table 2:** Concurrent/divergent validity indicators of the ETISR-SF.

	**ETISR-SF**	**PHQ-9**	**BAI**	**FAST**	**FNTD**
**ETISR-SF**	_ _ _	0.39**	0.46**	0.16	0.05
**General Trauma**	0.84**	0.35**	0.39**	0.11	-0.03
**Physical Abuse**	0.79**	0.26**	0.26*	0.15	-0.03
**Emotional Abuse**	0.75**	0.35**	0.47**	0.04	0.22
**Sexual Events**	0.53**	0.23*	0.29**	0.23*	0.14

* p=0.05; **p=0.01

It can be observed that the ETISR-SF correlated predominantly and moderately with the PHQ-9 and BAI scales, which respectively evaluate symptoms of depression and anxiety, indicating an interface between the constructs evaluated. In contrast, no correlation was observed between the SR-ETISR and the scales that assess substance abuse, except the correlations encountered between the experience of sexual traumas and the abusive consumption of alcohol. The moderate correlation seen between the different subscales (0.27 to 0.53), indicating the co-occurrence of different traumas in the same individual, should also be underscored.

### 5: Factor Structure

**Figure 1:**
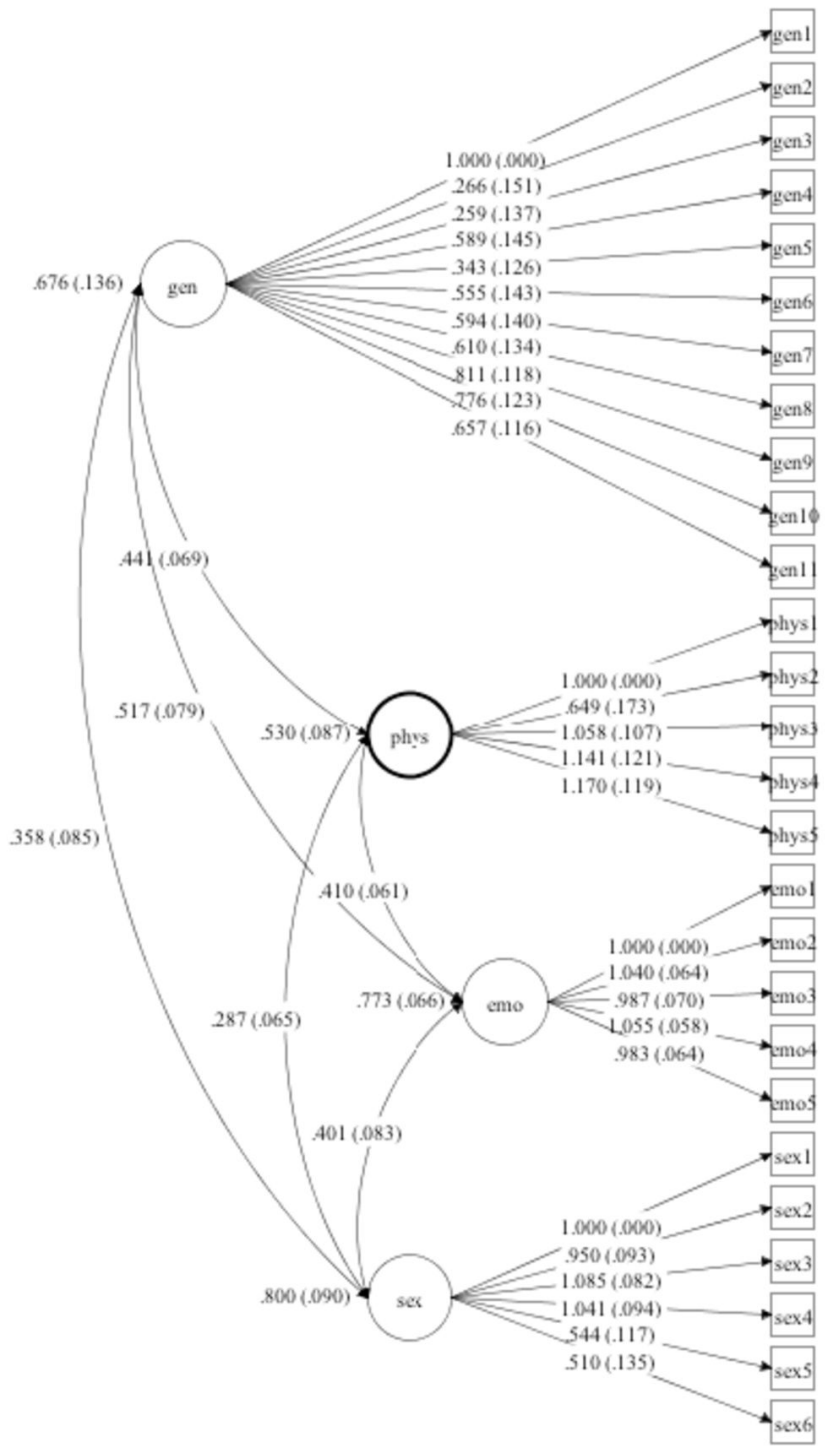
Correlated 4-factor Model.

**Figure 2:**
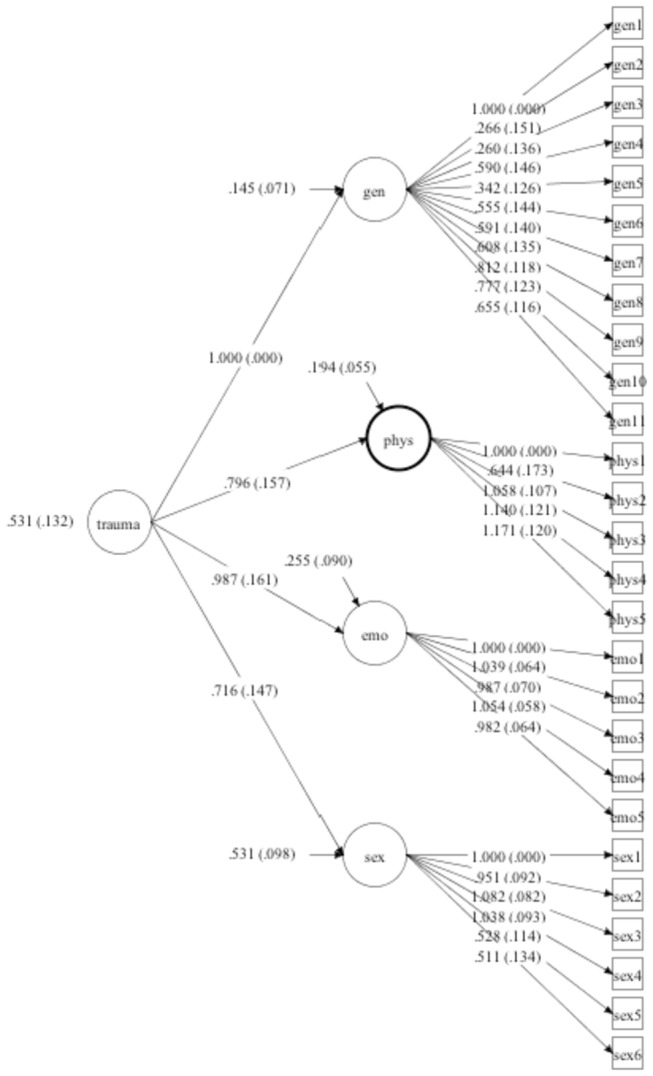
Second-order 4-factor Model.

Fit indexes from CFA are depicted in Table 3. A general trauma model with a single factor did not reveal adequate fit and suggested a unidimensional solution is not proper for the scale items. Also the bifactor model did not converged in our sample. Despite that, both the correlated four-factor model and the second order model with one high order trauma factor and four subsumed lower order latent factors revealed adequate fit indexes (Figures 1 and 2).

**Table 3:** Fit indexes for alternative models.

	**Free Parameters**		**X^2^**	**df**	**RMSEA**	**CI90%**		**CFI**	**TLI**	**WRMR**
**1-factor**		54	606.668	324	0.059	0.051	0.066	0.849	0.837	1.436
**Correlated 4-factor**		60	443.866	318	0.040	0.030	0.048	0.933	0.926	1.108
**Second-order 4-factor**		58	443.436	320	0.039	0.030	0.048	0.934	0.928	1.111
**Bifactor 4-factor**		Not-converged								

### 6: Test-retest reliability

A subset of 50 patients answered the questionnaires again a week after the first application. The intraclass correlation coefficient for the general scale and subscales ranged between 0.78 and 0.90.

### 7: Clinical indicators/ Discriminative Validity

Seeking a clinical analysis of the data and directing the study toward discriminative validity, the mean score of the subjects in the ETISR-SF was calculated, subdividing them into groups according to presence or absence of indicators of depression and anxiety. The mean ETISR-SF score found was 8.64 (SD =5.44) for the subjects with indicators of depression, while for the group without these indicators, the mean was 5.1 (SD =4.20), a statistically significant difference (U=680.00, p=0.004). For the subjects with anxiety indicators, the mean was 9.25 (SD =5.44) and for those without anxiety 4.48 (SD =3.9), also a significant difference from a statistical viewpoint (U=480.50, p<0.0001).

## Discussion

In general, the psychometric indicators of the ETISR-SF evaluated here in a sample of Brazilian subjects were found to be adequate. The translated version of the scale showed to be reliable in terms of both internal consistency and test-retest, showed an expected correlation with scores of emotional psychopathology (that are closely related to early traumatic experiences) and a factor structure that fits the original propositions.

It is noteworthy that in comparison with other samples that used ETISR-SF, this is a more ‘broad sample’, i.e., samples in these other studies are mostly composed of specific groups and those in vulnerable situations such as puerperae [23], depressive people, drug dependent people [22] and war veterans [21]. By observing the prevalence of early trauma through the item analysis, percentages were found very close to those reported by Anda et al. [1] and Dube et al. [2] who evaluated samples through an email questionnaire. Differences are encountered when these indices are compared to specific samples, as addressed by Rademaker et al [21] in the Netherlands. In this group, the percentage of emotional and sexual trauma was much lower than found here, indicating possible cultural differences.

The internal consistency of the ETISR-SF was adequate and in agreement with previous studies, even when evaluating the separate subscales [18,20–24]. In addition the good indices of reliability of the scale attest to its temporal stability.

Regarding concurrent validity, the correlation coefficient indices found between the ETISR-SF and these instruments, especially the PHQ-9 and the BAI were classified as moderate, in agreement with the clinical findings of the literature that indicate a high association between early traumas and symptoms from these psychiatric disorders [8,36-39]. This fact verifies, to a certain extent, the concurrent validity of the ETISR-SF, but also its divergent validity, as it differs from the specific aspects evaluated by these instruments. Conversely, no correlations were found with the scales of alcohol and tobacco abuse (substance abuse), except for the positive correlation between sexual trauma and alcohol abuse. However, several studies [40-42] frequently reported such correlations, but independent from the type of trauma. These studies also showed that the co-occurrence between these two situations also depends on the presence of other vulnerable situations, such as previous family psychiatric history, especially involving substance abuse, parental divorce, multiple traumas exposure, etc. The moderate correlations observed between the subscales also draw attention to a very important aspect from the clinical point of view: the co-occurrence of different “groups” of traumas in the same individual, indicating the high vulnerability of these subjects.

The factor structure reveal that both a correlated four factor solution and a second-order solution with 1 high order factor and 4 lower order factor showed adequate fit to the data. This provides support for the original model of the scale. The differences between the correlated and second-order structure have also theoretical implications. The correlated model implies a separate set of correlated risk factors linked to each type of traumatic events. A second-order model implies that the trauma has a different typology but a common etiological influence. Both models are somewhat supported by current theories, but further studies are needed with larger samples to better investigate the scale factor structure. In addition, other sensitive designs can shed light on the etiology of early traumatic experiences.

Our study has some limitations. First, the assessment of the severity of these traumatic experiences is a complex issue. This is because the evocation of memories of the traumatic childhood experiences in adult individuals is greatly influenced by the emotions associated with them, and permeated by distorted memories, blocked mnemonics, and affective cognitive dissociations, among others, factors that should be considered in the investigation of childhood trauma/abuse [3]. Second, to study the concurrent validity a scale specific for the evaluation of traumas was not used, which can be considered a limitation of the study. However, constructs closely related to trauma were used and confirmed positive associations among them.

In conclusion, the variety of indicators used here provides support for ETISR-SF as a reliable and valid instrument for assessing early traumatic experiences. Given the importance of trauma as a public health problem, tools such as ETISR-SF may help clinicians and researchers to better evaluate and measure such events and further advance clinical care of trauma victims.

## Supporting Information

Figure S1 **ETISR-SF - Brazilian version.**
(DOC)
