# Supplementary material for: Psychometrics Properties of Early Trauma Inventory Self Report – Short Form (ETISR-SR) for the Brazilian Context
Source: PLoS One. 2013 Oct 3;8(10):e76337. doi: 10.1371/journal.pone.0076337 (PMC3789732; doi:10.1371/journal.pone.0076337)
Supplement: Supplementary file 1 [file pone.0076337.s001.doc]

Inventário de Auto-Avaliação de Traumas Precoces - Versão Reduzida (ETISR-SF)

COPYRIGHT: J. Douglas Bremmer (2009). Tradução para o português do Brasil: Flávia de Lima Osório, Kátia Cruvinel Arrais, Ila Marques Porto Linares, João Paulo Machado de Sousa, Ana Vilela Mendes, José Alexandre de Souza Crippa (2010)

Identificação:____ Idade:________ Data da avaliação:____________

**Parte 1. Traumas Gerais. <u>Antes da idade de 18 anos</u>**			
1.	Você já foi exposto a algum desastre natural com ameaça a sua vida?	SIM	NÃO
2.	Você já se envolveu em um acidente grave?	SIM	NÃO
3.	Você já sofreu um ferimento ou uma doença grave?	SIM	NÃO
4.	Você já presenciou a morte ou alguma doença grave em seus pais ou responsáveis?	SIM	NÃO
5.	Você vivenciou o divórcio ou separação de seus pais?	SIM	NÃO
6.	Você já presenciou a morte ou um ferimento grave em um dos seus irmãos?	SIM	NÃO
7.	Você já presenciou a morte ou um ferimento grave em um amigo?	SIM	NÃO
8.	Você já presenciou situações de violência dirigida a outras pessoas, incluindo membros da sua família?	SIM	NÃO
9.	Alguém na sua família já sofreu de algum transtorno mental ou psiquiátrico, ou já teve um surto?	SIM	NÃO
10.	Os seus pais ou responsáveis já tiveram problemas com alcoolismo ou abuso de drogas?	SIM	NÃO
11.	Você já viu alguém assassinado?	SIM	NÃO
**Parte 2. Castigo Físico. <u>Antes da idade de 18 anos</u>**			
1.	Você já recebeu um tapa no rosto?	SIM	NÃO
2.	Você já foi queimado com água quente, cigarro ou alguma outra coisa?	SIM	NÃO
3.	Você já recebeu socos ou chutes?	SIM	NÃO
4.	Você já foi atingido por algum objeto arremessado contra você?	SIM	NÃO
5.	Você já foi empurrado?	SIM	NÃO
**Parte 3. Abuso Emocional. <u>Antes da idade de 18 anos</u>**			
1.	Você era frequentemente colocado para baixo ou ridicularizado?	SIM	NÃO
2.	Você era frequentemente ignorado ou tratado como se não fosse importante?	SIM	NÃO
3.	Frequentemente lhe diziam que você não era bom?	SIM	NÃO
4.	Na maioria das vezes você foi tratado com frieza, falta de carinho ou lhe fizeram sentir que não era amado?	SIM	NÃO
5.	Seus pais ou responsáveis frequentemente não conseguiam entender você ou suas necessidades?	SIM	NÃO
**Parte 4. Eventos Sexuais. <u>Antes da idade de 18 anos</u>**			
1.	Você já foi tocado em alguma parte íntima do seu corpo (como seios, coxas, genitais) de um jeito que te surpreendeu ou te fez sentir desconfortável?	SIM	NÃO
2.	Você já teve a experiência de alguém esfregar os genitais em você?	SIM	NÃO
3.	Você já foi forçado ou coagido a tocar em partes íntimas do corpo de outra pessoa?	SIM	NÃO
4.	Alguém já fez sexo com você contra a sua vontade?	SIM	NÃO
5.	Você já foi forçado ou coagido a fazer sexo oral em alguém contra a sua vontade?	SIM	NÃO
6.	Você já foi forçado ou coagido a beijar alguém de maneira sexual ao invés de um modo carinhoso?	SIM	NÃO

Se você respondeu "SIM" para qualquer um dos eventos acima, responda a seguir considerando o evento que teve o maior impacto em sua vida. Na sua resposta, considere como você se sentiu no momento do evento.

1.	Você experimentou medo intenso, pavor ou desamparo?	SIM	NÃO
2.	Você se sentiu fora do seu corpo ou como se você estivesse em um sonho?	SIM	NÃO
